# Systematic Evaluation of Genomic Prediction Algorithms for Genomic Prediction and Breeding of Aquatic Animals

**DOI:** 10.3390/genes13122247

**Published:** 2022-11-29

**Authors:** Kuiqin Wang, Ben Yang, Qi Li, Shikai Liu

**Affiliations:** 1 Key Laboratory of Mariculture, Ministry of Education, College of Fisheries, Ocean University of China, Qingdao 266003, China; 2 Laboratory for Marine Fisheries Science and Food Production Processes, Qingdao National Laboratory for Marine Science and Technology, Qingdao 266237, China

**Keywords:** genomic prediction, machine learning, aquatic animals, algorithm comparison, genomic selection

## Abstract

The extensive use of genomic selection (GS) in livestock and crops has led to a series of genomic-prediction (GP) algorithms despite the lack of a single algorithm that can suit all the species and traits. A systematic evaluation of available GP algorithms is thus necessary to identify the optimal GP algorithm for selective breeding in aquaculture species. In this study, a systematic comparison of ten GP algorithms, including both traditional and machine-learning algorithms, was conducted using publicly available genotype and phenotype data of eight traits, including weight and disease resistance traits, from five aquaculture species. The study aimed to provide insights into the optimal algorithm for GP in aquatic animals. Notably, no algorithm showed the best performance in all traits. However, reproducing kernel Hilbert space (RKHS) and support-vector machine (SVM) algorithms achieved relatively high prediction accuracies in most of the tested traits. Bayes A and random forest (RF) better prevented noise interference in the phenotypic data compared to the other algorithms. The prediction performances of GP algorithms in the *Crassostrea gigas* dataset were improved by using a genome-wide association study (GWAS) to select subsets of significant SNPs. An R package, “ASGS,” which integrates the commonly used traditional and machine-learning algorithms for efficiently finding the optimal algorithm, was developed to assist the application of genomic selection breeding of aquaculture species. This work provides valuable information and a tool for optimizing algorithms for GP, aiding genetic breeding in aquaculture species.

## 1. Introduction

Nowadays, selective breeding has become essential in the aquaculture industry. The growing demand for aquaculture products has led to an increased interest in improving productive and efficiency traits [1,2,3,4,5,6]. Traditional breeding approaches usually select individuals based only on phenotype, although molecular marker information is also used in some cases [1,4,5,6]. Breeding organizations around the world have been using the genomic selection (GS) approach in both crops and livestock [2,3,4]. In many species, GS achieves a higher selection response than pedigree-based selection (PBLUP) and marker-assisted selection (MAS) [5,6,7,8]. Genomic prediction (GP) refers to the prediction process of prediction accuracy during GS [9]. The application of GP requires breeding organizations to genotype a large number of marker loci using high-density marker arrays [10]. In recent years, GP has benefited from the advancement of the genotyping method by utilizing next-generation sequencing and SNP array platforms [11]. 

Several recent GP studies have been conducted in aquatic animals, mainly focusing on productive and efficiency traits, such as growth and disease resistance. GP potentially performs efficiently in aquatic animals because of the large number of offspring produced by a pair of parents and the use of high-density marker panels to genotype animals [12,13]. GP is also considered an ideal method for complex traits, such as growth traits controlled by many genetic loci with minor effects [14,15]. To date, GP-based studies in aquaculture have mainly focused on productive and efficiency traits [12]. Furthermore, GP for disease resistance has been conducted in large yellow croaker *(Larimichthys crocea)* [16], rainbow trout (*Oncorhynchus mykiss*) [17], hybrid red tilapia (*Oreochromis* spp.) [11], gilthead sea bream *(Sparus aurata)* [18], and Nile tilapia (*O. niloticus*) [19]. GP for growth-related traits has been performed in various species, such as Nile tilapia (*O. niloticus*) [20], Pacific white shrimp (*Litopenaeus vannamei*) [21], and Atlantic salmon (*Salmo salar*) [15]. The aforementioned studies proved that there are no best algorithms for all species and traits. Furthermore, studies that combined genome-wide association study (GWAS) with GP showed promising results. Studies has proved that GP can achieve higher prediction accuracy when using top SNPs with a significant *p*-value selected by GWAS [16,22].

Numerous algorithms have been proposed for GP. Each algorithm is usually suitable for one or more scenarios [23]. GP has faced the problem of “large p small n,” where the number of SNPs (p) is much larger than that of individuals (n) because of the rapid progress of next-generation sequencing [24,25], a phenomenon implying the data is highly-dimensional [26]. Non-additive effects have also been commonly observed in both plants and animals [26,27,28]. These lead to the proposition of non-parametric algorithms, including machine-learning methods, that deal with high-dimensionality data [26,29,30]. Machine-learning methods, such as reproducing kernel Hilbert space (RKHS), support-vector machine (SVM), gradient-boosting machine (GBM), random forest (RF), extreme gradient boosting (XgBoost), elastic net, adaptive boosting (AdaBoost), and deep learning, have already been used in selective-breeding programs in both crops and livestock [23,24,26,29,31,32,33]. It is therefore necessary to test the efficiency of these machine-learning methods when used in aquaculture scenarios. However, only a few studies related to the use of machine-learning methods have been performed in specific aquaculture species [34,35]. A systematic comparison of traditional and machine-learning algorithms for GP can thus provide valuable information to identify the optimal algorithm for specific traits in different aquaculture species.

In this study, ten genomic-prediction algorithms, including both traditional methods and machine-learning methods, were used to conduct a systematic comparison of publicly available genotype and phenotype data from five aquaculture species. This study aimed to (1) compare the genomic-prediction accuracy and computational time of traditional and machine-learning methods, (2) evaluate the prediction accuracy of different algorithms upon inclusion of noise from phenotypic data and using top SNPs with significant *p*-value selected by GWAS, and (3) develop an R package embedded with the commonly used traditional and machine-learning algorithms to assist in selecting the optimal algorithm for specific datasets. 

## 2. Materials and Methods

### 2.1. Datasets

Publicly available datasets from GP studies in five species, including the Pacific oyster (*Crassostrea gigas*) [36], Atlantic salmon (*S. salar*) [37], rainbow trout (*O. mykiss*) [17], Nile tilapia (*O. niloticus*) [20], and common carp (*Cyprinus carpio*) [38], were retrieved from the aforementioned articles for the analyses in this work. The *C. gigas* dataset was generated from a GWAS study aimed at assessing oyster resistance to *Ostreid herpesvirus* (OsHV-1). The *S. salar* dataset was generated from a GP study aimed at assessing genomic-prediction accuracy for salmon resistance to amoebic gill disease. The *O. mykiss* dataset was from a GP study aimed at assessing genomic-prediction accuracy for trout resistance to *Piscirickettsia salmonis.* The *C. carpio* dataset was from a GP study aimed at assessing genomic-prediction accuracy for the resistance of carp to koi herpesvirus disease (KHVD). The *O. niloticus* dataset was from a GP study aimed at assessing genomic-prediction accuracy for growth-related traits, including fillet yield and harvest weight. The missing genotypes of the aforementioned datasets were randomly imputed using the R package “synbreed” (version 0.12-9) [39]. Table 1 outlines the number of SNPs and individuals, and the traits analyzed in each dataset. Notably, the survival trait is expressed in binary format with 0 and 1 denoting dead and survivor individuals, respectively, whereas the mean gill and amoebic-load trait in the *S. salar* dataset and the weight traits in the *O. mykiss* dataset, *C. carpio* dataset, and *O. niloticus* dataset were continuous traits. Notably, the mean gill refers to how the resistance to amoebic gill disease is defined. It is the mean of the gill-lesion score recorded for both gills. The amoebic load is the qPCR values using *Neoparamoeba perurans* specific primers [37].

### 2.2. Evaluation of Traditional Genomic-Prediction Algorithms

Five traditional genomic-prediction algorithms, including Bayes A (BA), Bayes B (BB), Bayes C (BC), Bayesian ridge regression (BRR), and Bayesian lasso (BL), were fitted to the data, followed by an assessment of their prediction accuracy. The general formula of Bayes A and Bayes B is

(1) $$y_{i}=\mu +\sum_{j=1}^{h}\beta_{j}X_{\mathrm{ij}}+e$$

where i = 1… for the p individual, j = 1… for the h marker, y_i_ is the vector of phenotypes to the ith individual, μ stands for the vector of the constant term, β_j_ is the vector of regression coefficient to be estimated, X_ij_ is the incidence vector to individual i and marker j (0, 1, 2 stands for genotype AA, Aa, and aa), and e represents the random residual vector [2]. Bayes B assumed that only part of the markers contributed to the genetic variance, while Bayes A assumed that the variances of each marker had the same prior distribution. Bayes C belonged to the Bayes Cπ, where parameter π was set to 0.9 [31,40]. The Bayesian ridge-regression algorithm, a Bayesian version of RR-BLUP [3], was proposed by Hsiang [41] and applied in GP by Pérez and de los Campos [42], assuming that all markers have the same genetic variance [3]. The RR-BLUP is fundamentally equivalent to GBLUP [43], meaning that Bayesian ridge regression can represent RR-BLUP and GBLUP. The Bayesian lasso algorithm was used in GP [42,44] by adding a regularization parameter and assuming that the markers follow the Laplace distribution [3]. All five algorithms were implemented in the R package “BGLR” (version 1.1.0) [42]. The iterations were set at 12,000, burn-in at 2000, degree of freedom at 5, and thin at 5. The response-type parameter for binary phenotypes was set as “ordinal.” Supplementary Table S1 provides the example codes for the aforementioned traditional algorithms.

### 2.3. Evaluation of Machine-Learning Algorithms

Five machine-learning algorithms, including artificial neural network (ANN), reproducing kernel Hilbert space (RKHS), support-vector machine (SVM), gradient-boosting machine (GBM), and random forest (RF) were evaluated for their genomic-prediction performance. ANN belongs to neural network algorithms, RKHS and SVM belong to kernel-based algorithms, while GBM and RF belong to decision-tree algorithms [30,45].

ANN was implemented in the R package “brnn” (version 0.8) [46], fitting the simplest two-layer neural network, as described by Foresee and Hagan [47]. The algorithm described by Nguyen and Widrow [48] was used to assign initial weights, while the Gauss–Newton algorithm was used to perform the optimization. The “neurons” parameter in ANN highlighted the number of neurons in the hidden layer; a larger parameter significantly increased the complexity of the algorithm, thus increasing its computational time [49]. The RKHS algorithm was implemented in the R package “BGLR” (version 1.1.0) [42]. Notably, RKHS is a semi-parametric model replacing the genomic relationship matrix with the general kernel matrix to draw similarities between individuals despite being genetically uncorrelated [23,45]. The “h” parameter in RKHS is responsible for the shape of the probability density function in RKHS. A larger “h” smoothens the probability density function [50]. SVM was implemented in the R package “kernlab” (version 0.9-31) [51]. SVM was originally developed as a classifier and aimed to solve the separation problems of hyperplane having the largest geometric interval that can correctly divide a given training dataset [23,51]. The “epsilon” parameter indicates the tolerance where no penalty is given to constraint violation; a larger “epsilon” allows the existence of more errors. In the same line, the “C” parameter defines the cost of the violation; a larger “C” adds more penalties to the violation [51,52]. The RF algorithm was implemented through the R package “randomForest” (version 4.7-1.1) [53] and adopted a bootstrap resampling method to select a subset of observations to train numbers of decision trees each time. Each decision tree was grown using two-level randomization in the learning process, in which the new data result was decided by the voting score of each tree [26,54]. The “ntree” parameter defines the number of decision trees. However, the tree number should not be too small to achieve a higher prediction accuracy. The “nodesize” parameter defines the size of the trees to be grown. GBM was implemented using the R package “gbm” (version 2.1.8) [55]. It is an improvement on RF because the model in each iteration is updated and the resulting residuals are used to select the next model in a sequential manner [56]. Of note, the sampling of subsets in GBM depends on the weights of previous samples [26]. The “n.trees” parameter is the same as the “ntree” parameter in RF. The “shrinkage” parameter defines the learning rate; smaller learning rates commonly require more trees to be grown. The “interaction.depth” parameter defines the numbers of splits on each tree. A higher “interaction.depth” parameter thus increases the complexity of the GBM algorithm [55,57]. Notably, as the aforementioned hyperparameters significantly affect prediction accuracy, they were tuned to improve the prediction performances [34,58]. Table 2 outlines the hyperparameters tuned for each algorithm. Specifically, Supplementary Table S2 shows the value of hyperparameters set for the five machine-learning algorithms, while Supplementary Table S1 provides the example codes for the machine-learning algorithms.

### 2.4. Validation Methods 

The prediction accuracies of the continuous traits were evaluated using Pearson correlation coefficient between the observed phenotypes and genomic estimated breeding value (GEBV). The area under the curve (AUC) was used for survival traits (0 and 1 denoted dead and survivor individuals). Hold-out cross-validation was applied by randomly selecting 10% of the samples as the testing population while the remaining 90% represented the training population. The cross-validation was repeated 50 times, a correlation was obtained every time and then an average value of correlation was obtained. 

### 2.5. Effect of Noisy Phenotypic Data on Algorithm Prediction Accuracy

Biological experiments usually have uncontrolled environmental conditions. This, and the inherently unpredictable nature of individuals (for example, the distribution of alleles), lead to considerable amounts of noise [32]. Noisy data can affect the phenotypic data, reducing the accuracy of genomic prediction. Evaluating the effect of noisy phenotypic data on algorithm prediction performances is thus important. In this study, the effect of adding noise to phenotype data was investigated by randomly adding or subtracting twice the standard deviation of phenotype data to identify the most robust algorithm. The noise was gradually increased to 10%, 20%, 30%, 40%, 50%, 60%, and 70% to evaluate the prediction accuracy under different phenotypic noise ratios. The weight trait of *O. niloticus* was selected to conduct the study because it was the only dataset specializing in the weight trait. ANN was excluded from this analysis because it required high-quality datasets and cannot run adding noise. The cross-validation was repeated 50 times at each point, a correlation was obtained every time and then an average value of correlation was obtained.

### 2.6. Optimizing Prediction Accuracy by Using SNPs Selected by GWAS

In order to optimize the prediction accuracy of each algorithm in the *C. gigas* dataset, GWAS was performed through R package “GAPIT” (version 3.1.0) [59]. We used R package “BLINK” (version 0.1.0) to get markers with significant *p*-value [60]. The first three PCA were used to rectify population structure in GWAS [61]. The GP was conducted after sorting SNPs according to *p*-value results in GWAS. We gradually added the percent of SNPs from the best 10% to all, with 5% at a time, to evaluate prediction accuracy under different significant marker densities. The cross-validation was repeated 50 times at each point, a correlation was obtained every time and then an average value of correlation was obtained.

### 2.7. Evaluation of Computational Time

The survival trait in *O. mykiss* was used to compare the computational times when fitting each algorithm because it had a considerable number of individuals and thus would achieve more accurate computational times. The dataset contained phenotype and genotype data of 2047 individuals and 26,068 SNPs. No parallelization was adopted in these comparisons. All the algorithms were benchmarked using a single core of Intel^®^ Xeon^®^ Platinum 8164 CPU @ 2.00 GHz.

### 2.8. Hyperparameter Tuning in R Package “ASGS” for Machine-Learning Algorithms

An R package “ASGS” was developed to assist the efficient use of the GP algorithms, including the seven traditional (Bayes A, Bayes B, Bayes C, GBLUP, Bayesian lasso, Bayesian RR, and rrBLUP) and five machine learning (ANN, GBM, RF, RKHS, and SVM) algorithms. The hyperparameters in machine-learning algorithms can significantly influence their prediction accuracies [58]. An auto hyperparameter tuning function was thus compiled through the grid-search method for machine-learning algorithms to enhance their prediction accuracies. The training dataset in each cross-validation was used to tune the hyperparameters. The hyperparameters that significantly influenced the prediction accuracy (as described in Section 2.3 in the Materials and Methods) in the machine-learning algorithms were selected for auto hyperparameter tuning. Three groups of values were pre-set for each hyperparameter and were then subjected to all possible combinations for different hyperparameters. The validation method of the grid-search adopted the one described in Section 2.4 in the Materials and Methods. The combination of hyperparameters that achieved the highest prediction accuracy was used for the subsequent predictions. To evaluate the hyperparameter tuning function in R package “ASGS”, the survival trait in *C. gigas* was used to compare the AUC scores of tuning hyperparameters manually and automatically. The comparison was conducted under different significant marker densities.

## 3. Results

### 3.1. Prediction Accuracies of the Algorithms

**Figure 1:**
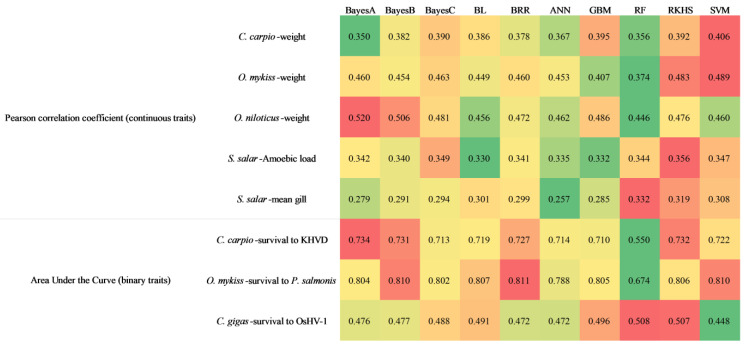
Prediction accuracies of the ten genomic-prediction algorithms analyzing various traits in five aquaculture species. The Pearson correlation coefficient and area under the curve (AUC) were used to evaluate the prediction accuracy of algorithms in each of the traits. Green denotes the lowest prediction accuracy in each trait, while red denotes the highest prediction accuracy.

Figure 1 shows the prediction accuracy evaluated by both Pearson correlation coefficient and AUC. The standard deviation is shown in Supplementary Table S1. Notably, eight traits in five species were used to conduct the comparison. Nearly all the algorithms showed higher prediction accuracy in one or more traits. Bayes A performed well in the weight trait of *O. niloticus* (0.520) and the resistance to KHVD trait of *C. carpio* (0.734). Bayes B performed well in the weight trait of *O. niloticus* (0.506), resistance to KHVD trait of *C. carpio* (0.731), and resistance to *P. salmonis* trait of *O. mykiss* (0.810). Both the Bayesian lasso and Bayesian ridge regression performed well in the resistance to *P. salmonis* trait of *O. mykiss* at 0.807 and 0.811, respectively. Moreover, GBM performed well in the weight trait of *C. carpio* (0.395), while RF performed well in the mean gill trait of *S. salar* (0.332). Of note, RKHS performed well in the weight trait of *C. carpio* (0.392), the weight trait of *O. mykiss* (0.483), the amoebic-load trait of *S. salar* (0.356), mean gill trait of *S. salar* (0.319), and resistance to KHVD trait of *C. carpio* (0.732). SVM performed well in the weight traits of *C. carpio* (0.406) and *O. mykiss* (0.489). Notably, RKHS and SVM achieved relatively high prediction accuracy in 5 out of 8 traits. Moreover, there was a significant difference between the poorly and well-performed algorithms in each trait. For instance, the highest prediction accuracy of the survival trait in *C. carpio* was 33% higher than the lowest prediction accuracy, while that of the amoebic-load trait in *S. salar* was 8%. Notably, all algorithms achieved AUC scores near 0.5 in the *C. gigas* dataset.

### 3.2. Prediction Accuracy of Algorithms Affected by Noise in Phenotypic Data

**Figure 2:**
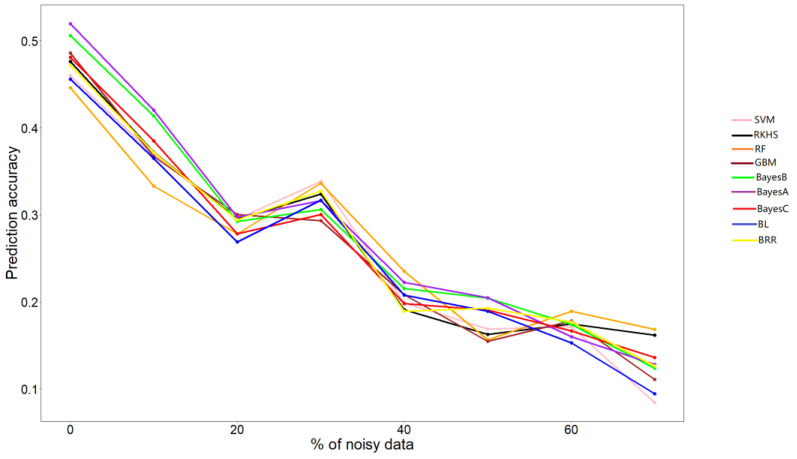
Prediction accuracy of each algorithm when noisy data is added to the phenotype. The *O. niloticus* weight-trait dataset was used. The percentage of noisy data included in the phenotype data and the prediction efficiency evaluated by Pearson correlation coefficient are represented in the X and Y axes, respectively.

All algorithms exhibited a declining trend in accuracy as the amount of noisy data increased (Figure 2). Well-performing algorithms significantly changed upon adding a different amount of noise to the phenotype data. Notably, Bayes A and RF performed better in more situations when noise was added. Bayes A outperformed the other algorithms when 10%, 40%, and 50% of noise were added. Similarly, RF outperformed the other algorithms when 30%, 40%, 60%, and 70% of noise were added. 

### 3.3. Prediction Efficiency of Algorithms in When Using SNPs Selected by GWAS

**Figure 3:**
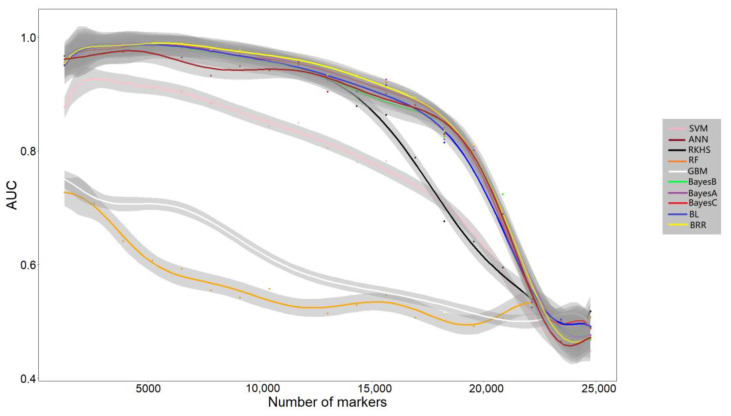
Prediction efficiency of each algorithm when using GWAS to select subsets of significant SNPs. The dataset of *C. gigas* was used. The number of markers used and the prediction efficiency evaluated by AUC (area under the curve) are represented in the X and Y axes, respectively. The grey area indicates 90% confidence interval.

The results are shown in Figure 3. Notably, all algorithms achieved higher AUC scores when using top SNPs compared to the case when using all SNPs. The highest AUC scores for most algorithms were achieved based on 4500 SNPs. All the algorithms achieved low AUC scores when using all SNPs in the *C. gigas* dataset. 

### 3.4. The Performance of Algorithms in Computational Time

**Figure 4:**
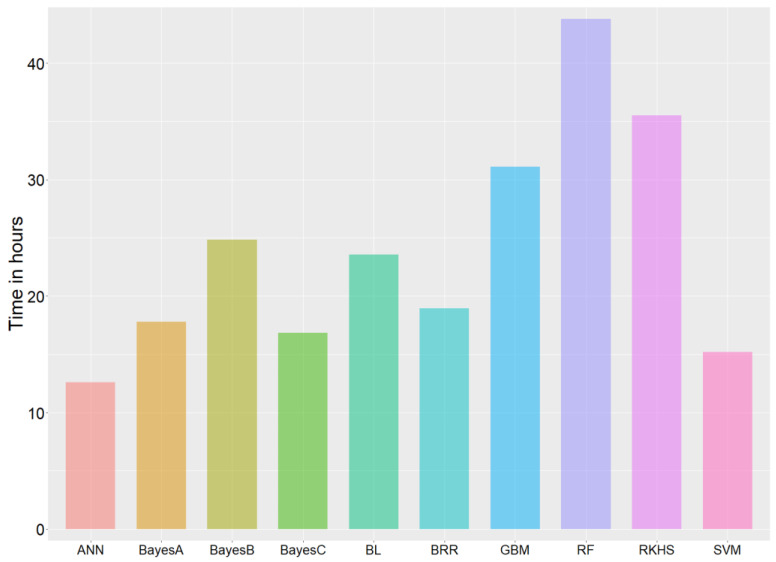
Comparison of the required computational time for fitting each of the ten algorithms. The *O. mykiss* survival trait dataset was used for this analysis.

Figure 4 shows the required computational time of 50 times cross-validation of each algorithm. The hyperparameters set for each machine-learning algorithm are shown in Supplementary Table S2. Notably, the computational time of different algorithms varied significantly. RF and GBM, both belonging to the decision-tree algorithm, were highly time-consuming, especially when the size of forest trees was large. ANN and SVM performed better than the time-consuming traditional Bayes algorithms and had comparable or even better prediction accuracies than the traditional algorithms in some traits. RKHS and SVM, the two optimal algorithms (Figure 1), varied significantly in computational time. The computational time of RKHS (approximately 35.5 h) was twice that of SVM (approximately 15.2 h). 

### 3.5. Development of R Package “ASGS”

**Figure 5:**
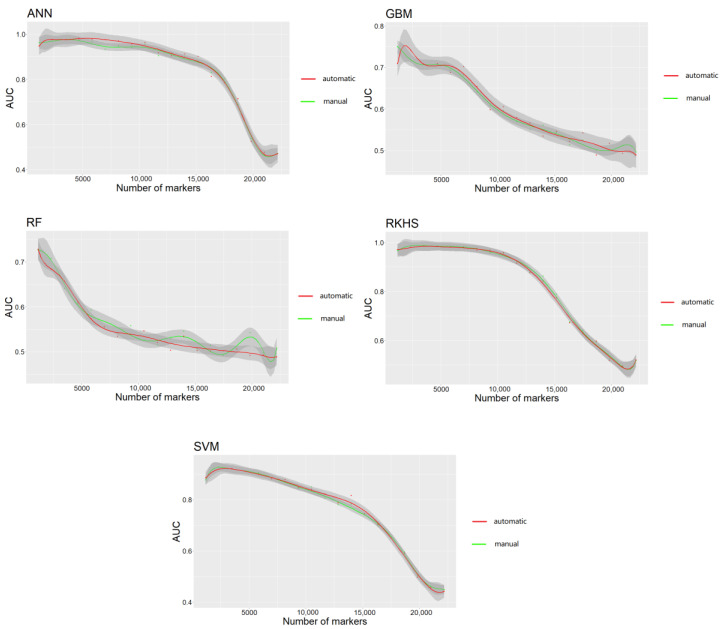
A comparison of AUC (area under the curve) scores of manual hyperparameter adjustment and automatic hyperparameter adjustment of five machine-learning algorithms. The *C. gigas* survival trait dataset was used for this analysis.

The R package “ASGS” was developed to assist in the efficient use of the GP algorithms. The input data comprised the genotypic, phenotypic, and fraction testing data. The 50 times cross-validation was implemented to obtain the mean prediction accuracy. The output data comprised a model-fitting situation and prediction accuracy for both the training and testing populations. Auto hyperparameter adjusting through the grid-search method was implemented for machine-learning algorithms to achieve a higher prediction accuracy. The R package “ASGS” can overcome the complex hyperparameter adjusting problem in the machine-learning algorithms. Figure 5 shows the comparison of five machine-learning algorithms when tuning the hyperparameters manually and using R package “ASGS”. Notably, the auto-hyperparameter-adjusting function achieved similar or even higher (in ANN and GBM) AUC scores compared to tuning hyperparameters manually. Users of the “ASGS” package in R will not have to adjust the hyperparameters independently. The “ASGS” package in R includes built-in example data using the *O. mykiss* dataset, as described earlier, to provide examples for users on how to run it. The package has been released to GitHub (https://github.com/Kuiqin/ASGS, accessed on 23 September 2022). 

## 4. Discussion

The extensive use of genomic selection in both livestock and crops has led to a series of algorithms, though none of the algorithms suit all the species and traits under study [58]. Studies that have applied GP algorithms in aquaculture species have mainly focused on comparison of traditional algorithms [62,63]. To date, no studies have compared the machine-learning algorithms with traditional algorithms in a variety of aquatic animals. A systematic assessment of available GP algorithms in aquatic animals is much needed. In this study, we retrieved publicly available genotype and phenotype data from five aquatic species to systematically compare ten GP algorithms, including both Bayesian and machine-learning methods. Notably, each algorithm outperformed others in one or more traits. Studies that have applied GP algorithms in livestock and crops also suggest that there is no best algorithm for every trait in all species [3,29,31,64,65]. 

Herein, RKHS and SVM achieved relatively high prediction accuracy in five out of eight traits, suggesting that RKHS and SVM are potentially optimal algorithms for more traits. Notably, SVM required much less computational time than RKHS. These findings collectively suggest that breeding organizations should try using SVM for less-well-known traits because of its relatively high prediction accuracy and reduced computational time. However, the prediction accuracy of any algorithm is affected by many factors, for example, the population structure and the degree of non-additive effects [31]. Breeding organizations should thus choose specific algorithms according to the characteristics of the data because each algorithm fits specific types of data structures. For example, Bayesian methods are suitable for traits more affected by dominant effects, while machine-learning methods are suitable for traits more affected by non-dominant effects [3,4,14,29,31,65].

Notably, all algorithms achieved low AUC scores (near 0.5) when using all SNPs in the *C. gigas* dataset. This phenomenon may be attributed to the small individual number and the existence of a large number of SNPs with no effects or negative effects. Therefore, using all SNPs to conduct GP is not suitable for the *C. gigas* dataset. Herein, we used the top SNPs with significant *p*-values selected by GWAS to conduct GP in the *C. gigas* dataset. The highest AUC scores for most algorithms were achieved based on 4500 SNPs. By reducing SNPs with no effects with negative effects, previous studies also proved that the prediction accuracy can be improved when using the top SNPs selected by GWAS [16,22]. 

Of note, the adjustment of the hyperparameters in machine-learning methods significantly affects the prediction accuracy and computational time (Materials and Methods 2.3 and the study conducted by Azodi [58]), while the traditional methods are unaffected. This phenomenon is attributed to the ability of the hyperparameters to affect the complexity of machine-learning algorithms. In this study, the “neurons” parameter in ANN, “ntree” in RF, and “n.trees” in GBM were positively correlated with the computational time [46,53,54,55,57]. For instance, the computational time for ANN lessened when the “neurons” parameter in ANN was set to 1. However, the computational demands of ANN grew tremendously when a larger “neurons” parameter was set. RF requires substantially less computational time when using Python library scikit-learn [34]. In contrast, RF proved to be highly time-consuming when using the R package “randomForest”. This phenomenon may be attributed to different RF implementation processes by the two packages. Moreover, RF appeared to be more time-consuming because the number of trees set in RF (1000) was larger than those in GBM (500). Notably, there is a certain degree of subjectivity in the argument of computational time because the number of iterations of the Markov chain Monte Carlo (MCMC) in the Bayesian algorithms varies depending on specific situations [34]. Machine-learning algorithms are promising in reducing computational time compared to the traditional Bayesian algorithms, especially when parallelization is used. 

Overfitting and underfitting are the two main causes of low prediction accuracy of a model. Overfitting is caused by the inclusion of too much useless information in the models, insufficient size of the training dataset, or the high complexity of the model. Underfitting is caused by a simplified and hence poorly fitted model to predict the multi-dimensional data sufficiently [66,67,68]. The complexity of machine-learning algorithms is also affected by their hyperparameters. For example, the “neurons” parameter in ANN positively correlates with the algorithm complexity [46]. To obtain a higher prediction accuracy, it is best to try to reduce the noise in the dataset and choose the most suitable algorithm according to the complexity of the training dataset. Breeding organizations should also choose different algorithms according to the data quality. For instance, decision-tree algorithms can achieve higher prediction accuracy in datasets that contain much noise. Notably, ANN could not function when noise was included, possibly because the dataset used was too small for training ANN when noise was included.

Numerous factors, including the number of individuals and SNPs, the degree of dominant effects and additive effects [4,14], and the existence of epistasis and environmental effects [69,70], can influence the prediction accuracy of an algorithm. Both traditional and machine-learning algorithms have their advantages in overcoming these situations. For instance, traditional algorithms are more likely to achieve a higher prediction accuracy when a trait is more affected by dominant effects [4,14]. Similarly, machine-learning algorithms tend to achieve a higher prediction accuracy in cases when epistasis and environmental effects are considered [69,70]. This study mainly focused on comparing GP algorithms in aquaculture species, without exploring the detailed characteristics of the various algorithms (for example, how the hyperparameters affect the structure and prediction accuracies of machine-learning algorithms). Future studies should therefore aim to explore more detailed characteristics of the algorithms, and benchmark the GP algorithms in cases of the existence of non-addictive effects. 

Notably, the adjustment of hyperparameters in machine-learning algorithms is quite time-consuming [58]. It has been shown that by using “ASGS” package in R, five machine-learning algorithms can achieve similar prediction accuracies compared to tuning the hyperparameters manually (Figure 5). Of note, there is a grid-search function in Python library scikit-learn [71]. However, there is no grid-search function in R. In order to assist the efficient use of the machine-learning algorithms in R, the grid-search function was compiled. The R package “ASGS” has combined the grid-search function with cross validation. When using the “ASGS” package in R, the hyperparameters will not have to be adjusted independently. Moreover, the “ASGS” package in R is user-friendly. Users will not have to write the codes because they have been encapsulated within the package.

In conclusion, none of the ten representative GP algorithms used to analyze datasets of eight traits in five species achieved the highest prediction accuracy in all the traits. However, RKHS and SVM achieved relatively high prediction accuracy in five out of eight traits, suggesting their suitability as the optimal algorithms for more traits. Notably, SVM required much less computational time than RKHS. The prediction accuracy of each algorithm was affected by the inclusion of noise in the phenotypic data. Bayes A and RF were the better algorithms when noise was included. The prediction accuracies of GP algorithms in *C. gigas* dataset were optimized by using GWAS to select subsets of significant SNPs. Furthermore, ANN and SVM outperformed the time-consuming traditional Bayes algorithms. The decision-tree algorithms and RKHS were proved to be highly time-consuming. The relationship between the complexity of the algorithms and data influences the prediction accuracy. Adapting complex algorithms to rather simple data causes overfitting, while adapting the simple algorithm to complex data causes underfitting. This work provides valuable information on the prediction efficiencies of the currently available GP algorithms and a useful tool for assisting in choosing the optimal algorithm for selective breeding of aquaculture species.

## Figures and Tables

**Table 1:** Datasets used in this study.

Species	Number of Markers	Number of Genotyped Individuals	Trait Analyzed	Data Sources (Accessed on 23 September 2022)
*Salmo salar*	16,797	1481	Amoebic load, mean gill	https://doi.org/10.1534/g3.118.200075
*Oncorhynchus mykiss*	26,068	2047	weight Resistance to *P. salmonis*	https://doi.org/10.1534/g3.119.400204
*Cyprinus carpio*	15,615	1260	Weight Resistance to KHVD	https://doi.org/10.3389/fgene.2019.00543
*Oreochromis niloticus*	32,306	1125	Weight	https://doi.org/10.1534/g3.119.400116
*Crassostrea gigas*	23,349	704	Resistance to OsHV-1	https://doi.org/10.1534/g3.118.200113

**Table 2:** Hyperparameters tuned in each algorithm.

Algorithm	Hyperparameters Tuned
ANN	neurons: the number of neurons.
GBM	Distribution: the distribution of the data used (squared error, absolute loss, and t-distribution loss, among others) n.trees: the total number of trees to fit shrinkage: a shrinkage parameter applied to each tree in the expansion, also known as the learning rate or step-size reduction interaction.depth: the maximum depth of each tree
RF	ntree: number of trees to grow mtry: Number of variables randomly sampled as candidates at each split nodesize: minimum size of terminal nodes
RKHS	h: bandwidth parameter
SVM	Kernel: the kernel function was used in training and predicting (radial basis, polynomial, Laplacian, and hyperbolic, among others) Epsilon: epsilon in the insensitive-loss function C: cost of constraints violation

## Data Availability

All datasets used in this study, including genotype and phenotype data, are openly available on GitHub (https://github.com/Kuiqin/DATA, accessed on 23 September 2022).

## Supplementary Materials

The following supporting information can be downloaded at: https://www.mdpi.com/article/10.3390/genes13122247/s1. Supplementary Table S1. Standard deviation of ten genomic-prediction algorithms analyzing various traits in five aquaculture species. Supplementary Table S2. Hyperparameters set for machine-learning algorithms analyzing various traits in five aquaculture species; p represents the number of markers. Supplementary Text S1. Example codes for fitting ten genomic-prediction algorithms.
