# Supplementary material for: Systematic Evaluation of Genomic Prediction Algorithms for Genomic Prediction and Breeding of Aquatic Animals
Source: Genes (Basel). 2022 Nov 29;13(12):2247. doi: 10.3390/genes13122247 (PMC9778314; doi:10.3390/genes13122247)
Supplement: Supplementary file 1 [file genes-13-02247-s001.zip › genes-1960725-supplementary/Supplementary Table .pdf]

Supplementary Table S1. Standard deviation of ten genomic prediction algorithms among various traits in five aquaculture species.

	BayesA	BayesB	BayesC	BL	BRR	ANN	GBM	RF	RKHS	SVM
C. carpio-weight	0.100	0.086	0.113	0.097	0.097	0.090	0.083	0.095	0.088	0.085
O. mykiss-weight	0.046	0.064	0.048	0.049	0.066	0.061	0.067	0.060	0.031	0.044
O. niloticus-weight	0.063	0.073	0.065	0.073	0.079	0.076	0.067	0.072	0.083	0.076
S. salar-Amoebic load	0.067	0.050	0.070	0.085	0.074	0.075	0.084	0.064	0.071	0.064
S. salar-mean gill	0.058	0.061	0.076	0.064	0.064	0.083	0.067	0.072	0.057	0.073
C. carpio-survival to KHVD	0.046	0.046	0.044	0.047	0.048	0.048	0.040	0.028	0.044	0.036
O. mykiss- survival to *P. salmonis*	0.030	0.026	0.029	0.026	0.027	0.035	0.031	0.030	0.029	0.026
C. gigas- survival to OsHV-1	0.015	0.014	0.014	0.011	0.013	0.011	0.007	0.009	0.005	0.019

Supplementary Table S2. Hyperparameters set for machine learning algorithms among various traits in five aquaculture species, p represents the number of markers.

	ANN	GBM	RF	RKHS	SVM
C. carpio-weight	neurons: 3	distribution: gaussian<br>n.trees: 500<br>shrinkage: 0.05<br>interaction.depth: 5	ntree: 500<br>mtry: p/3<br>nodesize: 5	h: 3	kernel: rbfdot<br>epsilon: 0.5<br>C: 2
O. mykiss-weight	neurons: 4	distribution: gaussian<br>n.trees: 500<br>shrinkage: 0.05<br>interaction.depth: 5	ntree: 500<br>mtry: p/3<br>nodesize: 2	h: 3	kernel: rbfdot<br>epsilon: 0.01<br>C: 0.5
O. niloticus-weight	neurons: 1	distribution: gaussian<br>n.trees: 1000<br>shrinkage: 0.06<br>interaction.depth: 5	ntree: 500<br>mtry: p/3<br>nodesize: 2	h: 3	kernel: rbfdot<br>epsilon: 0.1<br>C: 1
S. salar-Amoebic load	neurons: 2	distribution: gaussian<br>n.trees: 500<br>shrinkage: 0.05<br>interaction.depth: 1	ntree: 1000<br>mtry: p/3<br>nodesize: 5	h: 1.5	kernel: rbfdot<br>epsilon: 0.01<br>C: 0.1
S. salar-mean gill	neurons: 2	distribution: gaussian<br>n.trees: 1000<br>shrinkage: 0.025<br>interaction.depth: 3	ntree: 1000<br>mtry: p/3<br>nodesize: 2	h: 1.5	kernel: rbfdot<br>epsilon: 0.1<br>C: 0.5
C. carpio-survival to KHVD	neurons: 3	distribution: gaussian<br>n.trees: 1000<br>shrinkage: 0.025<br>interaction.depth: 1	ntree: 500<br>mtry: $\sqrt{p}$<br>nodesize: 2	h: 0.4	kernel: rbfdot<br>epsilon: 0.01<br>C: 2
O. mykiss- survival to *P. salmonis*	neurons: 1	distribution: gaussian<br>n.trees: 500<br>shrinkage: 0.05<br>interaction.depth: 5	ntree: 1000<br>mtry: $\sqrt{p}$<br>nodesize: 1	h: 1.5	kernel: rbfdot<br>epsilon: 0.05<br>C: 10
C. gigas- survival to OsHV-1	neurons: 2	distribution: gaussian<br>n.trees: 500<br>shrinkage: 0.1<br>interaction.depth: 3	ntree: 500<br>mtry: $\sqrt{p}$<br>nodesize: 1	h: 0.01	kernel: rbfdot<br>epsilon: 0.01<br>C: 1
