# Supplementary material for: Systematic Evaluation of Genomic Prediction Algorithms for Genomic Prediction and Breeding of Aquatic Animals
Source: Genes (Basel). 2022 Nov 29;13(12):2247. doi: 10.3390/genes13122247 (PMC9778314; doi:10.3390/genes13122247)
Supplement: Supplementary file 1 [file genes-13-02247-s001.zip › genes-1960725-supplementary/Supplementary Text. S1.pdf]

Supplementary Text. S1. Example codes for fitting ten genomic prediction algorithms.

```
#Fitting Bayes A
library(parallel)
library(doParallel)
  library(BGLR)
  library(ROCR)
X<-read.table('geno.txt',sep=' ',header=T)
Y0<-read.table('pheno.txt',sep=' ',header=T)
Y<-Y0[,6]
r<-0.1
correl<-numeric()
PMSE<-numeric()
aucre<-numeric()
cl <- makeCluster(10)
registerDoParallel(cl)

BA<-function(k){
co<-0;PMSE<-0;co1<-c();co2<-0;PMSE2<-0;auc2<-0;
yNA<-Y
tst<-sample(1:nrow(X),size=round(nrow(X)*r),replace=F)
yNA[tst]<-NA
  ETA<-list(list(X=as.matrix(X),model='BayesA',saveEffects=T))

  fm<-
BGLR(y=yNA,ETA=ETA,nIter=12000,burnIn=2000,df0=5,S0=NULL,saveAt='BayesA_',respons
e_type='ordinal')

co<-cor(as.numeric(fm$yHat[tst]),as.numeric(Y[tst]))
PMSE<-mean((as.numeric(fm$yHat[tst])-as.numeric(Y[tst]))^2)
  pred<-prediction(fm$yHat[tst],Y[tst])
  perf <- performance(pred,"tpr","fpr")
  auc <- performance(pred,'auc')
auc=unlist(slot(auc,"y.values"))
co2<-cor(as.numeric(fm$yHat[-tst]),as.numeric(Y[-tst]))
PMSE2<-mean((as.numeric(fm$yHat[-tst])-as.numeric(Y[-tst]))^2)
  pred<-prediction(fm$yHat[-tst],Y[-tst])
  perf <- performance(pred,"tpr","fpr")
  auc2 <- performance(pred,'auc')
auc2=unlist(slot(auc2,"y.values"))
co1<-c(co,PMSE,auc,co2,PMSE2,auc2)
return(co1)
}
```

```
system.time(results <-foreach(k =1:50,.combine = 'rbind',.packages =c('BGLR','ROCR')) %dopar% BA(k))

stopCluster(cl)
#get prediction accuracy and AUC
   Accuracy1<-mean(results[,1], na.rm=T)
   Accuracy2<-mean(results[,3], na.rm=T)
```

#Fitting BayesB

```
library(parallel)
library(doParallel)
  library(BGLR)
  library(ROCR)
X<-read.table('geno.txt',sep=' ',header=T)
Y0<-read.table('pheno.txt',sep=' ',header=T)
Y<-Y0[,6]
r<-0.1
correl<-numeric()
PMSE<-numeric()
aucre<-numeric()
cl <- makeCluster(10)
registerDoParallel(cl)

BB<-function(k){
co<-0;PMSE<-0;co1<-c();co2<-0;PMSE2<-0;auc2<-0;
yNA<-Y
tst<-sample(1:nrow(X),size=round(nrow(X)*r),replace=F)
yNA[tst]<-NA
  ETA<-list(list(X=as.matrix(X),model='BayesB',saveEffects=T))

fm<-
BGLR(y=yNA,ETA=ETA,nIter=12000,burnIn=2000,df0=5,S0=NULL,saveAt='BayesB_',respons
e_type='ordinal')

co<-cor(as.numeric(fm$yHat[tst]),as.numeric(Y[tst]))
PMSE<-mean((as.numeric(fm$yHat[tst])-as.numeric(Y[tst]))^2)
  pred<-prediction(fm$yHat[tst],Y[tst])
  perf <- performance(pred,"tpr","fpr")
  auc <- performance(pred,'auc')
auc=unlist(slot(auc,"y.values"))
co2<-cor(as.numeric(fm$yHat[-tst]),as.numeric(Y[-tst]))
PMSE2<-mean((as.numeric(fm$yHat[-tst])-as.numeric(Y[-tst]))^2)
  pred<-prediction(fm$yHat[-tst],Y[-tst])
```

```
  perf <- performance(pred,"tpr","fpr")
  auc2<- performance(pred,'auc')
auc2=unlist(slot(auc2,"y.values"))
co1<-c(co,PMSE,auc,co2,PMSE2,auc2)
return(co1)
}
system.time(results <-foreach(k =1:50,.combine = 'rbind',.packages =c('BGLR','ROCR')) %dopar% BB(k))

stopCluster(cl)
#get prediction accuracy and AUC
   Accuracy1<-mean(results[,1], na.rm=T)
   Accuracy2<-mean(results[,3], na.rm=T)
```

#Fitting Bayes C

```
library(parallel)
library(doParallel)
  library(BGLR)
  library(ROCR)
X<-read.table('geno.txt',sep=' ',header=T)
Y0<-read.table('pheno.txt',sep=' ',header=T)
Y<-Y0[,6]
r<-0.1
correl<-numeric()
PMSE<-numeric()
aucre<-numeric()
cl <- makeCluster(10)
registerDoParallel(cl)

BC<-function(k){
co<-0;PMSE<-0;co1<-c();co2<-0;PMSE2<-0;auc2<-0;
yNA<-Y
tst<-sample(1:nrow(X),size=round(nrow(X)*r),replace=F)
yNA[tst]<-NA

  ETA<-list(list(X=as.matrix(X),model='BayesC',saveEffects=T))

fm<-
BGLR(y=yNA,ETA=ETA,nIter=12000,burnIn=2000,df0=5,S0=NULL,saveAt='BayesC_',response_type='ordinal')

co<-cor(as.numeric(fm$yHat[tst]),as.numeric(Y[tst]))
PMSE<-mean((as.numeric(fm$yHat[tst])-as.numeric(Y[tst]))^2)
  pred<-prediction(fm$yHat[tst],Y[tst])
```

```
  perf <- performance(pred,"tpr","fpr")
  auc <- performance(pred,'auc')
auc=unlist(slot(auc,"y.values"))
co2<-cor(as.numeric(fm$yHat[-tst]),as.numeric(Y[-tst]))
PMSE2<-mean((as.numeric(fm$yHat[-tst])-as.numeric(Y[-tst]))^2)
  pred<-prediction(fm$yHat[-tst],Y[-tst])
  perf <- performance(pred,"tpr","fpr")
  auc2 <- performance(pred,'auc')
auc2=unlist(slot(auc2,"y.values"))
co1<-c(co,PMSE,auc,co2,PMSE2,auc2)
return(co1)
}
system.time(results <-foreach(k =1:50,.combine = 'rbind',.packages =c('BGLR','ROCR')) %dopar% BC(k))

stopCluster(cl)
#get prediction accuracy and AUC
    Accuracy1<-mean(results[,1], na.rm=T)
    Accuracy2<-mean(results[,3], na.rm=T)
```

#Fitting BL

```
library(parallel)
library(doParallel)
  library(BGLR)
  library(ROCR)
X<-read.table('geno.txt',sep=' ',header=T)
Y0<-read.table('pheno.txt',sep=' ',header=T)
Y<-Y0[,6]
r<-0.1
correl<-numeric()
PMSE<-numeric()
aucre<-numeric()
cl <- makeCluster(10)
registerDoParallel(cl)

BL<-function(k){
co<-0;PMSE<-0;co1<-c();co2<-0;PMSE2<-0;auc2<-0;
yNA<-Y
tst<-sample(1:nrow(X),size=round(nrow(X)*r),replace=F)
yNA[tst]<-NA

  ETA<-list(list(X=as.matrix(X),model='BL',saveEffects=T))

fm<-
```

```
BGLR(y=yNA,ETA=ETA,nIter=12000,burnIn=2000,df0=5,S0=NULL,saveAt='BL_',response_ty
pe='ordinal')

co<-cor(as.numeric(fm$yHat[tst]),as.numeric(Y[tst]))
PMSE<-mean((as.numeric(fm$yHat[tst])-as.numeric(Y[tst]))^2)
  pred<-prediction(fm$yHat[tst],Y[tst])
  perf <- performance(pred,"tpr","fpr")
  auc <- performance(pred,'auc')
auc=unlist(slot(auc,"y.values"))
co2<-cor(as.numeric(fm$yHat[-tst]),as.numeric(Y[-tst]))
PMSE2<-mean((as.numeric(fm$yHat[-tst])-as.numeric(Y[-tst]))^2)
  pred<-prediction(fm$yHat[-tst],Y[-tst])
  perf <- performance(pred,"tpr","fpr")
  auc2 <- performance(pred,'auc')
auc2=unlist(slot(auc2,"y.values"))
co1<-c(co,PMSE,auc,co2,PMSE2,auc2)
return(co1)
}
system.time(results <-foreach(k =1:50,.combine = 'rbind',.packages
=c('BGLR','ROCR')) %dopar% BL(k))

stopCluster(cl)
#get prediction accuracy and AUC
    Accuracy1<-mean(results[,1], na.rm=T)
    Accuracy2<-mean(results[,3], na.rm=T)

#Fitting BRR
library(parallel)
library(doParallel)
  library(BGLR)
  library(ROCR)
X<-read.table('geno.txt',sep=' ',header=T)
Y0<-read.table('pheno.txt',sep=' ',header=T)
Y<-Y0[,6]
r<-0.1
correl<-numeric()
PMSE<-numeric()
aucre<-numeric()
cl <- makeCluster(10)
registerDoParallel(cl)

BRR<-function(k){
co<-0;PMSE<-0;co1<-c();co2<-0;PMSE2<-0;auc2<-0;
yNA<-Y
```

```
tst<-sample(1:nrow(X),size=round(nrow(X)*r),replace=F)
yNA[tst]<-NA

  ETA<-list(list(X=as.matrix(X),model='BRR',saveEffects=T))

fm<-
BGLR(y=yNA,ETA=ETA,nIter=12000,burnIn=2000,df0=5,S0=NULL,saveAt='BRR_',response_t
ype='ordinal')

co<-cor(as.numeric(fm$yHat[tst]),as.numeric(Y[tst]))
PMSE<-mean((as.numeric(fm$yHat[tst])-as.numeric(Y[tst]))^2)
  pred<-prediction(fm$yHat[tst],Y[tst])
  perf <- performance(pred,"tpr","fpr")
  auc <- performance(pred,'auc')
auc=unlist(slot(auc,"y.values"))
co2<-cor(as.numeric(fm$yHat[-tst]),as.numeric(Y[-tst]))
PMSE2<-mean((as.numeric(fm$yHat[-tst])-as.numeric(Y[-tst]))^2)
  pred<-prediction(fm$yHat[-tst],Y[-tst])
  perf <- performance(pred,"tpr","fpr")
  auc2 <- performance(pred,'auc')
auc2=unlist(slot(auc2,"y.values"))
co1<-c(co,PMSE,auc,co2,PMSE2,auc2)
return(co1)
}
system.time(results <-foreach(k =1:50,.combine = 'rbind',.packages
=c('BGLR','ROCR')) %dopar% BRR(k))

stopCluster(cl)
#get prediction accuracy and AUC
    Accuracy1<-mean(results[,1], na.rm=T)
    Accuracy2<-mean(results[,3], na.rm=T)
```

#Fitting ANN

```
library(brnn)
library(ROCR)
X<-read.table('geno.txt',sep=' ',header=T)
Y0<-read.table('pheno.txt',sep=' ',header=T)
Y<-Y0[,6]
r<-0.1
    n<-nrow(X)
    p<-ncol(X)
    XS<-as.matrix(X)

    for(i in 1:ncol(XS)){ (XS[,i]<-XS[,i]-mean(XS[,i]))/sd(XS[,i])}
```

```
   G<-tcrossprod(XS)/ncol(XS)
   correl<-numeric()
aucre<-numeric()
system.time(for(k in 1:50){
      tst<-sample(1:n,size=round(n*r),replace=F)

      GTRN<-G[-tst,] ; YTRN<-Y[-tst]
      GTST<-G[tst,] ; YTST<-Y[tst]

      NN<-brnn(y=YTRN,x=GTRN,neurons=2, verbose=F)

      Pred1<- predict(NN, newdata=GTST)
Pred2<- predict(NN, newdata=GTRN)

      correl[k]<-cor(Pred1,YTST)
PMSE[k]<-mean((Pred1-YTST)^2)
  pred<-prediction(Pred1,YTST)
  perf <- performance(pred,"tpr","fpr")
  auc <- performance(pred,'auc')
  auc=unlist(slot(auc,"y.values"))
  aucre[k]<-auc
   })
#get prediction accuracy and AUC

   Accuracy1<-mean(correl, na.rm=T)
   Accuracy2<-mean(aucre, na.rm=T)
```

#Fitting RKHS

```
library(BGLR)
library(ROCR)
X<-read.table('geno.txt',sep=' ',header=T)
Y0<-read.table('pheno.txt',sep=' ',header=T)
y<-Y0[,6]
r<-0.1
h<-3
prediction<-numeric();
aucre<-numeric()
D<-as.matrix(dist(X,method="euclidean"))^2
  D<-D/mean(D)
system.time(for(i in 1:50){
print(paste('the',i,'time'))
yNA<-y
tst=sample(1:nrow(X),round(r*nrow(X)),replace=F)
yNA[tst]<-NA
```

```
K<-exp(-h*D)
ETA<-list(list(K=K,model='RKHS'))
prefix<- paste(h[i], "_",sep="")
fm<-BGLR(y=yNA,ETA=ETA,
                  nIter=12000,burnIn=2000,df0=5,S0=NULL,saveAt=prefix)
        prediction[i]<-cor(fm$yHat[tst],y[tst])
#auc
pred<-prediction(fm$yHat[tst],y[tst])
perf <- performance(pred,"tpr","fpr")
  auc <- performance(pred,'auc')
auc=unlist(slot(auc,"y.values"))
  aucre[i]<-auc
})
#get prediction accuracy and AUC

Accuracy1<-mean(prediction, na.rm=T)
Accuracy2<-mean(aucre, na.rm=T)
```

#Fitting GBM

```
library(gbm)
library(parallel)
library(doParallel)
library(ROCR)
X<-read.table('geno.txt',sep=' ',header=T)
Y0<-read.table('pheno.txt',sep=' ',header=T)
Y<-Y0[,6]
r<-0.1
correl<-numeric()
aucre<-numeric()
cl <- makeCluster(10)
registerDoParallel(cl)

GBM<-function(k){
co<-0;PMSE<-0;co1<-c();co2<-0;PMSE2<-0;auc<-0;auc2<-0;
tst<-sample(1:nrow(X),size=round(nrow(X)*r),replace=F)
XS<-scale(X,center=T,scale=T)
XTRN<-XS[-tst,] ; YTRN<-Y[-tst]
XTST<-XS[tst,] ; YTST<-Y[tst]
fm<-
gbm.fit(x=XTRN,y=YTRN,distribution="gaussian",shrinkage=0.05,n.trees=500,interaction.de
pth=5)
Predic<-predict(fm,XTST)
Predic2<-predict(fm,XTRN)
```

```
co<-cor(as.numeric(Predic),as.numeric(YTST))
PMSE<-mean((as.numeric(Predic)-as.numeric(YTST))^2)
#auc
pred<-prediction(as.numeric(Predic),as.numeric(YTST))
  perf <- performance(pred,"tpr","fpr")
  auc <- performance(pred,'auc')
auc=unlist(slot(auc,"y.values"))

co2<-cor(as.numeric(Predic2),as.numeric(YTRN))
PMSE2<-mean((as.numeric(Predic2)-as.numeric(YTRN))^2)
#auc2
pred<-prediction(as.numeric(Predic2),as.numeric(YTRN))
  perf <- performance(pred,"tpr","fpr")
  auc2 <- performance(pred,'auc')
auc2=unlist(slot(auc2,"y.values"))
co1<-c(co,PMSE,auc,co2,PMSE2,auc2)
return(co1)
}

system.time(results <-foreach(k =1:50,.combine = 'rbind',.packages =c('gbm','ROCR')) %dopar% GBM(k))

stopCluster(cl)

#get prediction accuracy and AUC
    Accuracy1<-mean(results[,1], na.rm=T)
    Accuracy2<-mean(results[,3], na.rm=T)
```

#Fitting RF

```
library(randomForest)
library(parallel)
library(doParallel)
library(ROCR)
X<-read.table('geno.txt',sep=' ',header=T)
Y0<-read.table('pheno.txt',sep=' ',header=T)
Y<-Y0[,6]
r<-0.1
correl<-numeric()
aucre<-numeric()
cl <- makeCluster(10)
registerDoParallel(cl)
```

```
RF<-function(k){
co<-0;PMSE<-0;co1<-c()
tst<-sample(1:nrow(X),size=round(nrow(X)*r),replace=F)
XS<-scale(X,center=T,scale=T)
XTRN<-XS[-tst,] ; YTRN<-Y[-tst]
XTST<-XS[tst,] ; YTST<-Y[tst]
Pred1<-randomForest(x=XTRN, y=YTRN, xtest=XTST,
ytest=YTST,importance=T,proximity=T,nodesize=2)
Predic<-Pred1$test$predicted
co<-cor(as.numeric(Predic),as.numeric(YTST))
PMSE<-mean((as.numeric(Predic)-as.numeric(YTST))^2)
pred<-prediction(as.numeric(Predic),as.numeric(YTST))
  perf <- performance(pred,"tpr","fpr")
  auc <- performance(pred,'auc')
auc=unlist(slot(auc,"y.values"))
co1<-c(co,PMSE,auc)
return(co1)
}

system.time(results <-foreach(k =1:50,.combine = 'rbind',.packages
=c('randomForest','ROCR')) %dopar% RF(k))

stopCluster(cl)

#get prediction accuracy and AUC
   Accuracy1<-mean(results[,1], na.rm=T)
   Accuracy2<-mean(results[,3], na.rm=T)
```

#Fitting SVM

```
library(kernlab)
library(ROCR)
X<-read.table('geno.txt',sep=' ',header=T)
Y0<-read.table('pheno.txt',sep=' ',header=T)
Y<-Y0[,6]
r<-0.1
n<-nrow(X)
   p<-ncol(X)
   correl<-numeric();aucre<-numeric();
   system.time(for (k in 1:50){
tst<-sample(1:n,size=round(n*r),replace=F)
       XTRN<-X[-tst,] ; YTRN<-Y[-tst]
       XTST<-X[tst,] ; YTST<-Y[tst]
```

```
        fm<-ksvm(y=YTRN,x=as.matrix(XTRN),type="eps-
svr",kernel="rbfdot",scale=T,epsilon=0.05,C=1)

        Pred1<-predict(fm,XTST,type="response")
print(paste('the',k,'time','accuracy=',cor(Pred1,YTST)))

        correl[k]<-cor(Pred1,YTST)
  pred<-prediction(Pred1,YTST)
  perf <- performance(pred,"tpr","fpr")
  auc <- performance(pred,'auc')
  auc=unlist(slot(auc,"y.values"))
  aucre[k]<-auc

    })
  #get prediction accuracy and AUC

    Accuracy1<-mean(correl, na.rm=T)
    Accuracy2<-mean(aucre, na.rm=T)
```
